# USING NCI-AD FOR LONG-TERM SERVICES AND SUPPORTS AGING AND DISABILITY SYSTEM IMPROVEMENT

**DOI:** 10.1093/geroni/igae098.1941

**Published:** 2024-12-31

**Authors:** Stephanie Giordano, Rosa Plasencia

**Affiliations:** Human Services Research Institute, Cambridge, Massachusetts, United States; ADvancing States, Arlington, Virginia, United States

## Abstract

National Core Indicators Aging and Disabilities (NCI-AD™), survey data support State Medicaid, aging, and disability agencies to conduct surveys with individuals receiving publicly-funded long-term services and supports and measure and track their own outcomes and performance using reliable data collection methods and tools. Established in 2015, NCI-AD is now in use by 23 states nationwide. The NCI-AD portfolio includes the flagship survey – the Adult Consumer Survey (ACS) and the State of the Workforce—Aging and Disabilities (SotW-AD) survey. The ACS collects data directly from older adults and people with physical disabilities to assess the outcomes of services provided to individuals. Indicators address key areas of concern including service planning, rights, community inclusion, choice, health and care coordination, safety and relationships. In 2022, NCI-AD piloted the SotW-AD is a first of its kind for the aging and disabilities field, this new effort collects and aggregates statewide information about wages, benefits, and turnover of the direct service workforce—as reported by the agencies that employ them. This session will provide an overview of the NCI-AD program, the type of data collected from each survey, and how state-level and research partners have utilized the data sets. Additionally, 18 NCI-AD measures are included in the newly released CMS HCBS Quality Measure Set. We will discuss how this impacts states, and potential ways that these and other measures will be implemented and used to support important aspects of services access and equity.

